# Supramolecular construction of a cyclobutane ring system with four different substituents in the solid state

**DOI:** 10.1038/s42004-021-00493-3

**Published:** 2021-05-10

**Authors:** Michael A. Sinnwell, Ryan H. Groeneman, Benjamin J. Ingenthron, Changan Li, Leonard R. MacGillivray

**Affiliations:** 1 Department of Chemistry, University of Iowa, Iowa City, IA USA; 2 Department of Biological Sciences, Webster University, St. Louis, MO USA

**Keywords:** Crystal engineering, Self-assembly

## Abstract

Methods to form cyclobutane rings by an intermolecular [2 + 2] cross-photoreaction (CPR) with four different substituents are rare. These reactions are typically performed in the liquid phase, involve multiple steps, and generate product mixtures. Here, we report a CPR that generates a cyclobutane ring with four different aryl substituents. The CPR occurs quantitatively, without side products, and without a need for product purification. Generally, we demonstrate how face-to-face stacking interactions of aromatic rings can be exploited in the process of cocrystallization and the field of crystal engineering to stack and align unsymmetrical alkenes in CPRs to afford chiral cyclobutanes with up to four different aryl groups via binary cocrystals. Overall, we expect the process herein to be useful to generate chiral carbon scaffolds, which is important given the presence of four-membered carbocyclic rings as structural units in biological compounds and materials science.

## Abstract (web summary)

Preparation of cyclobutanes with four different substituents is rare and often arduous. Here a cocrystallisation strategy enables the intermolecular [2+2] cross-photoreaction of non-symmetrical stilbene derivatives to obtain chiral tetrasubstituted cyclobutanes with up to four different substituents in quantitative yield.

## Introduction

**Fig. 1:**
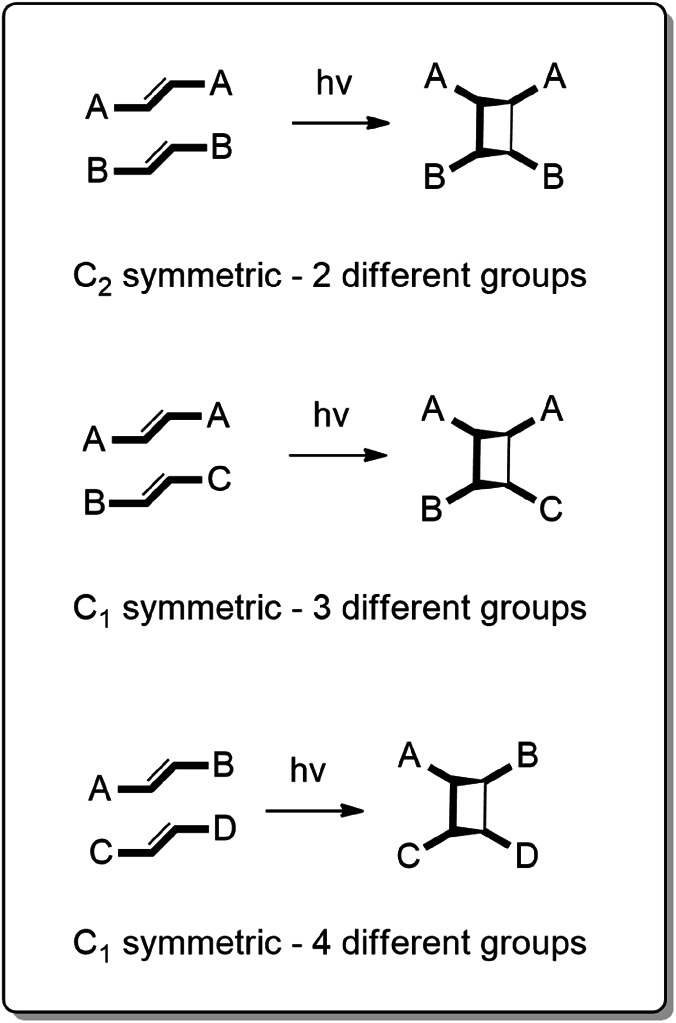
Cross-photoreactions involving different substituents to afford chiral cyclobutanes. Unsymmetric olefins can provide access to cyclobutanes bearing four different substituents.

Recent years have witnessed efforts to perform organic syntheses that are efficient, atom economical, and sustainable^1^. In this context, the ability to achieve an intermolecular [2 + 2] cross-photoreaction (CPR) to form a four-membered cyclobutane ring system with four different substituents—vis a vis a stereogenic carbon atom^2^—that occurs quantitatively and without side products represents an important and fundamental synthetic challenge (Fig. 1)^3–5^. In such a reaction, two unsymmetrical carbon–carbon double (C=C) bonds on two different molecules are expected to be assembled and preorganized parallel and in close proximity to photoreact.

Alkenes functionalized with perfluorophenyl (i.e., fully fluorinated) groups, along with phenyl-, and/or carboxylic acid groups, are known to undergo rare CPRs in the solid state^6^ upon cocrystallization^7–12^. For a photocycloaddition to occur in the solid state, two alkenes are expected to crystallize in a parallel arrangement with the C=C bonds separated on the order of 4.2 Å^13^. While the CPRs involving the perfluorophenyl groups^6^ have generated aryl-substituted cyclobutanes with up to three different substituents^6,12^, the formation of a cyclobutane ring with four different substituents has remained elusive. CPRs also occur in the solid state in statistical mixtures of solid solutions^14–16^, although an example of two different unsymmetrical alkenes assembling in a cocrystal that react in a CPR to form a cyclobutane with four different aryl groups quantitatively and without side products has not been reported.

**Fig. 2:**
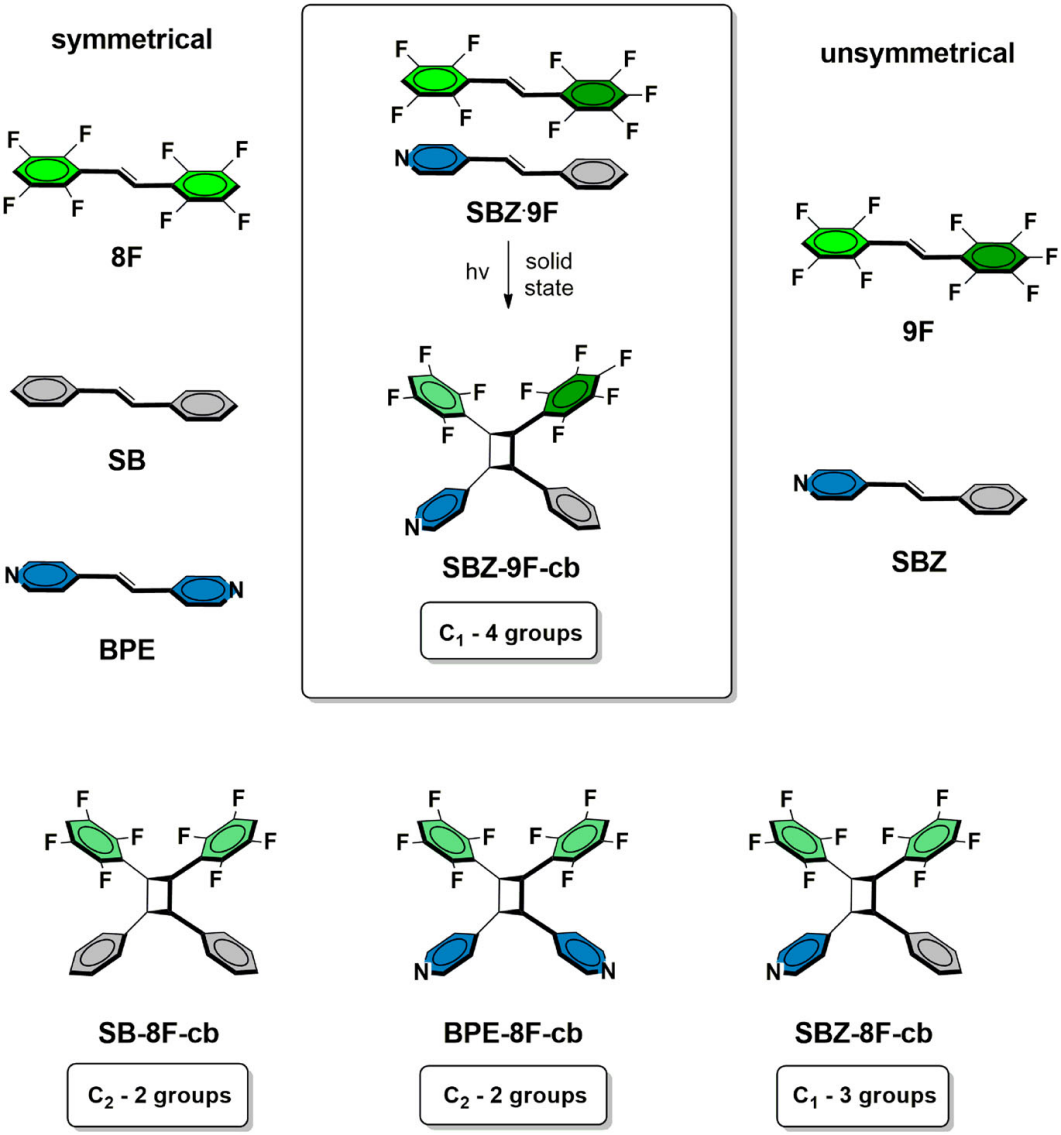
Cross-photoreactions studied to generate a cyclobutane with up to four different substituents. Perfluorophenyl-phenyl and H-perfluorophenyl-pyridyl stacking directs the assembly of C=C bonds.

**Fig. 3:**
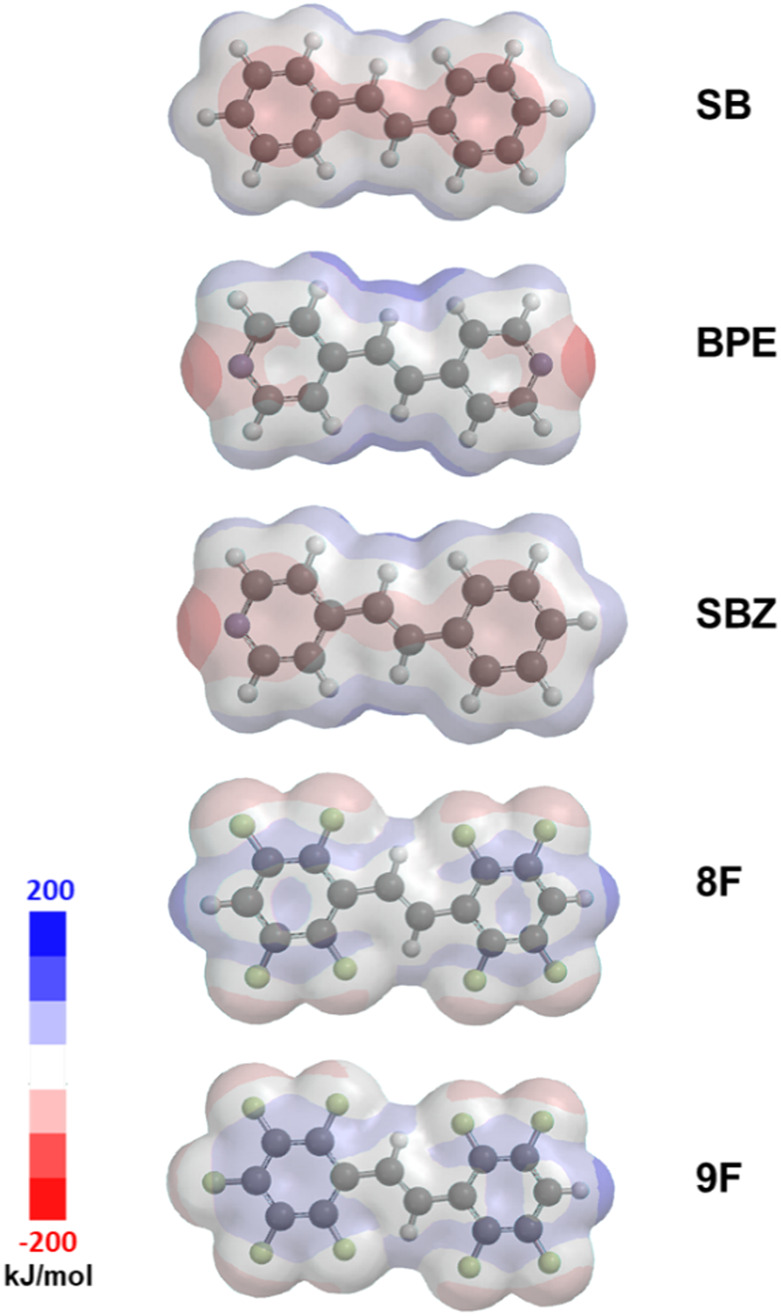
DFT calculated electrostatic map for functionalized alkenes (blue = positive electrostatic charge, red = negative electrostatic charge). Note terminal ends of **9F** to exhibit opposite charges.

Our solution to the aforementioned problem is to use four different functional groups that support two different face-to-face stacking interactions; namely, perfluorophenyl-phenyl and H-perfluorophenyl-pyridyl stacking to direct the assembly of C=C bonds to react (Fig. 2)^13^. We hypothesized that the partially fluorinated phenyl groups present in the symmetrical alkene *trans*-1,2-bis(2,3,5,6-tetrafluorophenyl)ethylene (**8F**) would have a capacity to participate in face-to-face stacking with phenyl groups, akin to a fully fluorinated analog^17^. Density functional theory (DFT) calculations demonstrated bond polarization and significant partial positive charges on the ring C-atoms of the C–F bonds of **8F** (Fig. 3). Such polarization supports stacking of fully fluorinated groups via perfluorophenyl-phenyl forces, and we expected the idea could be applied to the partially fluorinated analog **8F**^18^. DFT calculations also showed the end C–H groups to be polarized, having—in contrast—partial negative and positive charges on the C- and H-atoms, respectively. The fluorophenyl-bearing alkenes symmetrical **8F** and unsymmetrical *trans*-1-(2,3,5,6-tetrafluorophenyl)-2-(2,3,4,5,6-pentafluorophenyl)ethylene (**9F**) were, thus, used. Symmetrical *trans*-stilbene (**SB**) and unsymmetrical *trans*-4-stilbazole (**SBZ**) were used as the phenyl-bearing alkenes. Symmetrical *trans*-1,2-bis(4-pyridyl)ethylene (**BPE**) was also a reactant.

## Results and discussion

**Fig. 4:**
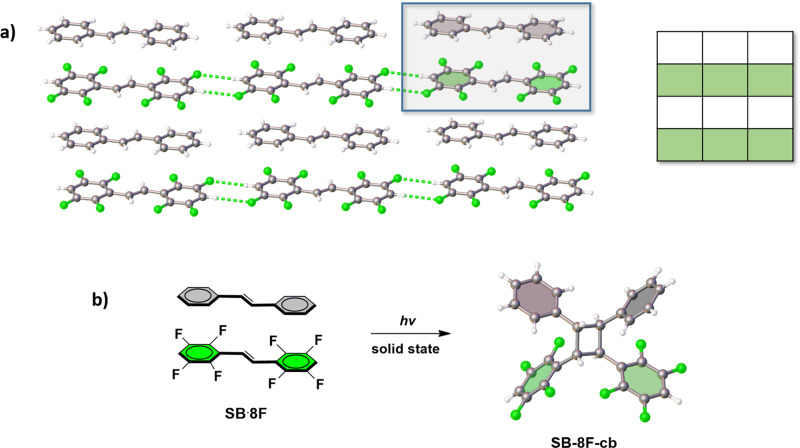
Cross-photoreaction showing X-ray structures. **a** **SBˑ8F** (inset: representation of packing type). **b** **SB-8F-cb** with solid-state photoreaction highlighted.

The symmetrical and partially fluorinated alkene **8F** was prepared by a Wittig reaction. When **8F** (25 mg, 0.077 mmol) in toluene (2.0 mL) was cocrystallized with **SB** (14 mg 0.077 mmol) in toluene (2.0 mL), colorless plate-shaped single crystals of the binary cocrystal **SB·8F** formed upon slow evaporation after a period of 2 days. The components of **SB·8F** crystallize in the centrosymmetric triclinic space group P ī (Table 1, Fig. 4) (Supplementary Data 1). The two alkenes participate in offset face-to-face π-stacking to form stacked columns, with **8F** of adjacent columns aligned end-to-end and interaction by means of C–H···F forces (Fig. 4a). Within each column, **SB** and **8F** stack in an alternating fashion and in registry^17^. The C=C bonds of the stacked molecules lie parallel and separated by 3.82 Å (centroid···centroid), which conforms to the criteria of Schmidt. When the cocrystal **SB·8F** was exposed to UV-radiation (medium-pressure Hg lamp) for a period of 50 h, the two alkenes reacted in an intermolecular CPR to form **SB**-**8F-cb** (chiral C_2_ symmetric) in quantitative yield. Cyclobutane formation was evidenced by the disappearance and appearance of the multiplets at 7.94 and 7.61 ppm, respectively.

**Table 1:** Crystallographic parameters for cocrystals that undergo CPRs.

	SB·8F	BPE·8F	SBZ·8F	SBZ·9F
Crystal system	Triclinic	Triclinic	Triclinic	Triclinic
Space group	*P* ī	*P* ī	*P* ī	*P* ī
*a* (Å)	6.2588(4)	6.286(3)	6.2524(8)	6.165(4)
*b* (Å)	7.6417(6)	7.690(4)	7.6581(12)	7.736(5)
*c* (Å)	11.6953(8)	11.654(7)	23.133(3)	23.677(14)
*α* (°)	87.276(4)	81.58(4)	87.316(9)	85.441(4)
*β* (°)	89.359(4)	81.41(4)	85.878(9)	87.457(4)
*γ* (°)	82.433(4)	81.23(4)	81.898(9)	82.052(4)
*V* (Å^3^)	553.86(7)	546.1(5)	1093.0(3)	1114.2(12)

The stereochemistry of **SB-8F-cb** was confirmed by single-crystal X-ray diffraction (Fig. 4b) (Supplementary Data 2). When the as-photoreacted powder was dissolved in toluene (1.0 mL) and ethanol (1.0 mL), colorless block crystals formed, upon slow evaporation. The cyclobutane product **SB-8F-cb** crystallizes in the triclinic space group P ī to form a layered structure within the crystallographic *ab*-plane. The centrosymmetric space group is consistent with the photodimerization affording a mixture of two enantiomers in equal amounts from the reacting solid. The cyclobutane exhibits a tongue-in-groove fit sustained by offset face-to-face H-perfluorophenyl-phenyl forces (3.88 Å).

**Fig. 5:**
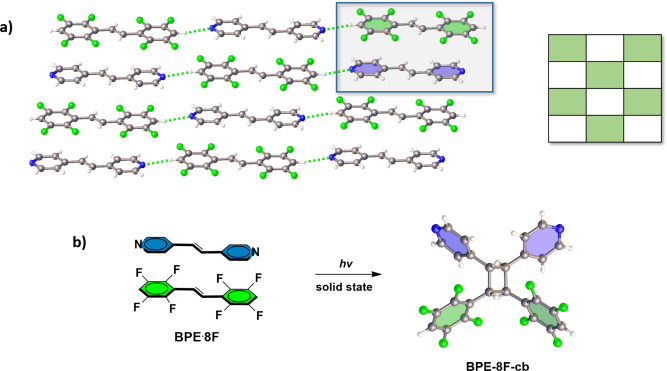
Cross-photoreaction showing X-ray structures. **a** **BPEˑ8F** (inset: representation of packing type), **b** **BPE-8F-cb** with solid-state photoreaction highlighted.

A CPR of **8F** was also achieved with the symmetrical bipyridine **BPE**. When **8F** (25 mg, 0.077 mmol) in toluene (2.0 mL) was cocrystallized with **BPE** (14 mg, 0.077 mmol) in toluene (1.0 mL) and ethanol (1.0 mL), colorless plate-shaped single crystals of **BPE·8F** formed upon slow evaporation after a period of 2 days. The components of **BPE·8F** also crystallize in the centrosymmetric triclinic space group P ī (Table 1, Fig. 5) with the alkenes in offset face-to-face π-stacking geometries (Supplementary Data 3). The C=C bonds of the stacked molecules are parallel and separated by 3.85 Å (Fig. 5a). Both end pyridyl N-atoms of **BPE** also participate in rare C–H···N type hydrogen bonds (3.30 Å) with **8F** to afford one-dimensional (1D) arrays. Thus, in contrast to **SB·8F**, the 1D columns are offset. The 1D arrays assemble such that the alkenes are also engaged in alternating face-to-face stacks. When the cocrystal **BPE·8F** was exposed to UV-radiation for a period of 50 h, **BPE-8F-cb** (chiral C_2_ symmetric) formed in quantitative yield as evidenced by the disappearance and appearance of the multiplets at 7.94 and 7.61 ppm, respectively. The stereochemistry of **BPE-8F-cb** was also confirmed by single-crystal X-ray diffraction with the cyclobutane crystallizing in the centrosymmetric space group P ī (Fig. 5b) (Supplementary Data 4).

**Fig. 6:**
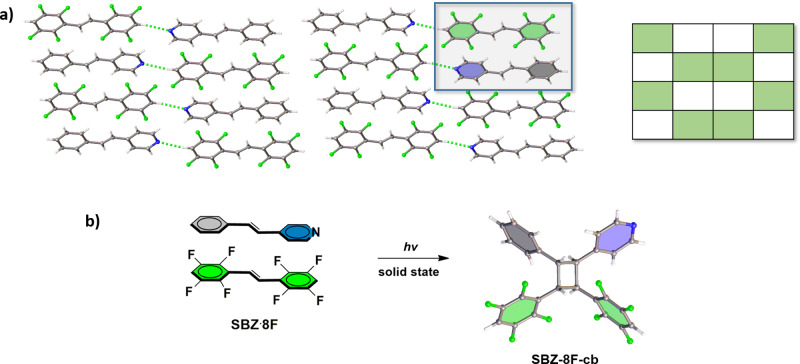
Cross-photoreaction showing X-ray structures. **a** **SBZˑ8F** (inset: representation of packing type), **b** **SBZ-8F-cb** with solid-state photoreaction highlighted.

The method also generates a cyclobutane ring with three different substituents. When symmetrical **8F** (25 mg, 0.077 mmol) was cocrystallized with unsymmetrical **SBZ** (14 mg, 0.077 mmol) in toluene (1.0 mL) and ethanol (1.0 mL), colorless rectangular-shaped single crystals of the binary cocrystal **SBZ·8F** formed upon slow evaporation after a period of 2 days. The components of **SBZ·8F** crystallize in the centrosymmetric triclinic space group P ī with the alkenes face-to-face (Table 1, Fig. 6) (Supplementary Data 5). The C=C bonds lie parallel, and separated by two unique distances of 3.81 and 3.85 Å (Fig. 6a). The olefins stack with the aromatic rings engaged in a combination of H-fluorophenyl-phenyl and H-fluorophenyl-pyridyl forces. The pyridyl N-atoms of **SBZ** also participate in C–H···N type hydrogen bonds (3.28 Å) with **8F** to define a two-component complex of offset columns. When **SBZ·8F** was exposed to UV-radiation for a period of 50 h, **SBZ-8F-cb** (chiral C_1_ symmetric) formed in quantitative yield. The stereochemistry of **SBZ-8F-cb** was confirmed by single-crystal X-ray diffraction with the cyclobutane crystallizing in the centrosymmetric triclinic space group P ī (Fig. 6b) (Supplementary Data 6).

Whereas stacking of fully fluorinated aromatic rings with phenyl groups is known, we are unaware of an example that uses partially fluorinated rings to achieve such face-to-face stacking in a cocrystal. Moreover, that the rings of **8F** participate in stacks with both a phenyl- and a pyridyl-group prompted us to turn to form a cyclobutane ring with four different substituents. We expected that cocrystallization of the unsymmetrical alkenes **9F** and **SBZ** would generate a binary cocrystal wherein the two alkenes assemble in a face-to-face stacked geometry. While two orientations of the stacked alkenes would be possible, perfluorophenyl-phenyl stacking was expected to be favored based on complementary electrostatics^19^. A cocrystallization of **9F** and **SBZ** would, thus, afford a binary cocrystal with the olefins stacked face-to-face (Fig. 2). A topochemical [2 + 2] photodimerization would generate the chiral cyclobutane **SBZ-9F-cb** (chiral C_1_ symmetric) that bears four chemically different substituents.

**Fig. 7:**
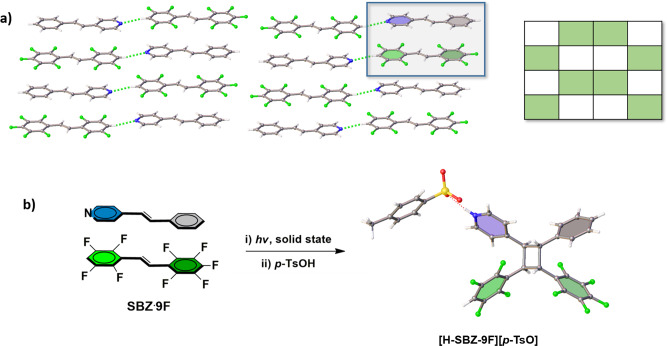
Cross-photoreaction showing X-ray structures. **a** **SBZˑ9F** (inset: representation of packing type), **b** [**H-SBZ-9F-cb**][*p*-**TsO**] with solid-state photoreaction and isolation as salt highlighted. Note four different groups present on cyclobutane ring system.

**Fig. 8:**
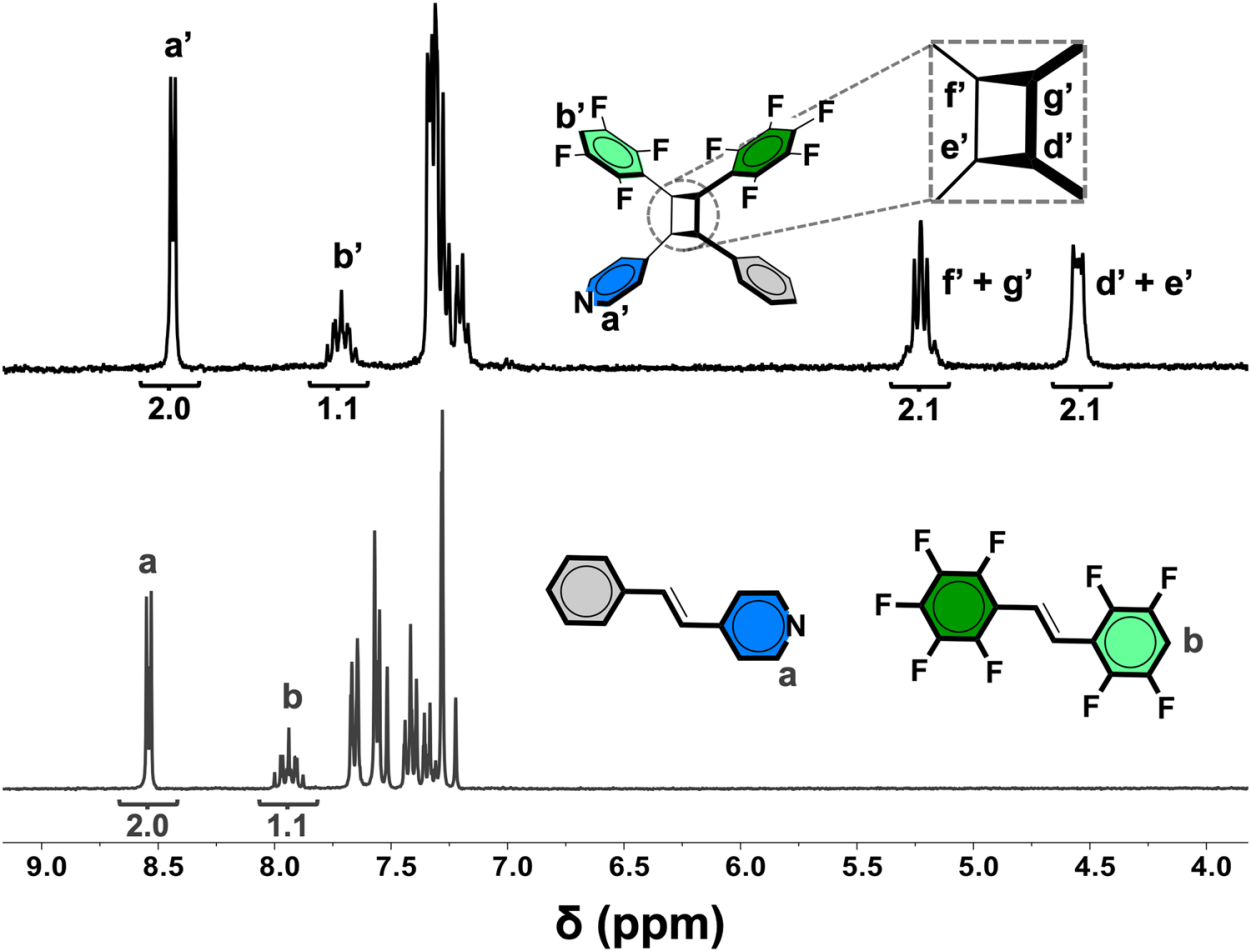
NMR spectroscopy. ^1^H NMR spectrum of photoreacted solid showing cross-photoreaction of **SBZ·9F** that generates cyclobutane **SBZ-9F-cb** with four different substituents (100% yield, no purification).

The unsymmetrical alkene **9F** was prepared by a Wittig reaction. When **9F** (25 mg, 0.073 mmol) in toluene (2.0 mL) was cocrystallized with unsymmetrical **SBZ** (13 mg, 0.073 mmol) in toluene (1.0 mL) and ethanol (1.0 mL), colorless plate-shaped single crystals of the binary cocrystal **SBZ·9F** formed upon slow evaporation after a period of 2 days. The components of **SBZ·9F** crystallize in the centrosymmetric triclinic space group P ī (Tables 1 and 2, Fig. 7) with the alkenes in offset face-to-face π-stacked geometries (Supplementary Data 7). The C=C bonds lie parallel and separated by two unique distances of 3.79 and 3.90 Å. The olefins stack with the aromatic rings engaged in a combination of perfluorophenyl-phenyl and H-perfluorophenyl-pyridyl forces. The end pyridyl N-atoms of **SBZ**, as in **BPE·8F**, participate in C–H···N hydrogen bonds (3.30 Å) to form two-component complexes of offset columns (Fig. 7a). The perfluoronated rings of **9F** also interact between columns by F···F forces (2.94 Å). When **SBZ·9F** was exposed to UV-radiation for a period of 50 h, the cyclobutane with four different aryl substituents **SBZ-9F-cb** (chiral C_1_ symmetric) formed in quantitative yield. The formation of **SBZ-9F-cb** was evidenced by the disappearance and appearance of the alkene and cyclobutane resonances, respectively (Fig. 8).

**Table 2:** Selected parameters of photoreactive cocrystals and cyclobutanes of CPRs.

	SB·8F	BPE·8F	SBZ·8F	SBZ·9F
C–H···N (Å)	−	3.30	3.28	3.30
C=C (Å)	3.821	3.85	3.81	3.91
CPR yield (%)	100%	100%	100%	100%
No. of different subsitutents	2	2	3	4
Cyclobutane symmetry	C_2_	C_2_	C_1_	C_1_

The stereochemistry of **SBZ-9F-cb** was confirmed by single-crystal X-ray diffraction (Fig. 7b) (Supplementary Data 8). Specifically, while **SBZ-9F-cb** resisted crystallization from a number of different organic solvents, reaction of **SBZ-9F-cb** with *p*-toluenesulfonic acid in dichloromethane-methanol (2 mL, 1:1, v:v) afforded single crystals of the salt [**H-SBZ-9F-cb**][*p*-TsO]. The components crystallize in the centrosymmetric monoclinic space group P2_1_/n. The X-ray data confirm attachment of the four different substituents and stereochemistry of the cyclobutane ring system of **SBZ-9F-cb**. The components assemble to form a channel-type solid with the cyclobutanes forming ribbons along the crystallographic *a*-axis.

In this report, we have demonstrated how principles of crystal engineering and supramolecular chemistry can be used to construct a cyclobutane with four different substituents. The cyclobutane photoproduct, along with other generated chiral cyclobutanes, forms quantitatively, without side products, and without a need for purification. The process is remarkable, given the facile manner in which all products form compared to liquid phase approaches. In experiments to further widen the scope, we expect the process to be useful to generate additional carbon scaffolds that are relevant to biologically important compounds and materials science. These ideas are also important to further efforts that focus to transiently trap compounds and control chemical reactivity ^20^.

## Methods

### Chemicals

Triphenylphosphine (Strem) 2,3,5,6-tetrafluorobenzyl bromide (Oakwood), 2,3,4,5,6-pentafluorobenzyl bromide (Oakwood), 2,3,5,6-tetrafluorobenzaldehyde (Oakwood), 2,3,4,5,6-pentafluorobenzaldehyde (Oakwood), sodium hydride (dry, Sigma-Aldrich), **BPE** (Sigma-Aldrich), **SB** (ACROS), and solvents (toluene, chloroform, ethanol, dimethylformamide) (Fisher) were commercially available and used as received.

### Photoreactions

All photochemical reactions were conducted using UV-radiation from a 450 W medium-pressure mercury lamp in an ACE Glass photochemistry cabinet. All cocrystals were finely ground using a mortar and pestle, and then placed between a pair of Pyrex glass plates. Samples were irradiated in 6-h intervals.

### Syntheses

For the syntheses and characterizations of triphenyl-(2,3,5,6-tetrafluorobenzyl)phosphonium bromide, **8F**, **SBZ**, and **9F**, as well as all cocrystals and products of photoreactions and CCDC codes (2042036-2042043), see Supplementary Methods.

## Supplementary information

Supplementary Data 1

Supplementary Data 2

Supplementary Data 3

Supplementary Data 4

Supplementary Data 5

Supplementary Data 6

Supplementary Data 7

Supplementary Data 8

Supplementary Information

Description of Additional Supplementary Files

## Data Availability

The X-ray crystallographic coordinates for structures of **SB**·**8F**, **SB-8F-cb**, **BPE**·**8F**, **BPE-8F-cb**, **SBZ**·**8F**, **SBZ-8F-cb**, **SBZ**·**9F**, and [**H**-(**SBZ-9F-cb**)][**p-TsO**] reported in this article have been deposited at the Cambridge Crystallographic Data Center (CCDC), under deposition numbers CCDC 2042036 to 2042043, respectively. These data can be obtained free of charge from The Cambridge Crystallographic Data Center via https://summary.ccdc.cam.ac.uk/structure-summary-form. Details of syntheses, NMR spectral data, and molecular modeling are provided in the Supplementary Material. All relevant data are available from the authors.
