# Supplementary material for: Supramolecular construction of a cyclobutane ring system with four different substituents in the solid state
Source: Commun Chem. 2021 May 10;4:60. doi: 10.1038/s42004-021-00493-3 (PMC9814370; doi:10.1038/s42004-021-00493-3)
Supplement: Supplementary file 9 — Supplementary Information [file 42004_2021_493_MOESM9_ESM.pdf]

Supporting Information for

Supramolecular construction of a cyclobutane ring system with four different substituents in the solid state

Michael A. Sinnwell, Ryan H. Groeneman,[‡]* Benjamin J. Ingenthron, Changan Li, and Leonard R. MacGillivray[†]*

[†]Department of Chemistry, University of Iowa, Iowa City, IA 65804, USA

[‡]Department of Biological Sciences, Webster University, St. Louis, MO 63119, USA

S1. Syntheses and Photoreactions Pages S2-S3

S2. ^{1}H NMR Spectral Data Pages S4-S11

S3. Powder X-ray Diffraction Data Pages S12-S16

S4. Single Crystal X-ray Diffraction Data Pages S17-S21

S5. Molecular Modeling Calculations Pages S22-S23

S6. Supplementary References Pages S24

S1. Syntheses and Photoreactions

Supplementary Methods

Triphenyl-(2,3,5,6-tetrafluorobenzyl)phosphonium bromide,[1-2] **8F**,[1-2] and **SBZ**[3] were synthesized according to literature. Triphenylphosphine (Strem) 2,3,5,6-tetrafluorobenzyl bromide (Oakwood), 2,3,4,5,6-pentafluorobenzyl bromide (Oakwood), 2,3,5,6-tetrafluorobenzaldehyde (Oakwood), 2,3,4,5,6-pentafluorobenzaldehyde (Oakwood), sodium hydride (dry, Sigma-Aldrich), **BPE** (Sigma-Aldrich), **SB** (ACROS), and solvents [toluene, chloroform, ethanol, dimethylformamide] (Fisher) were commercially available and used as received. The cross photoreactions were conducted using UV-radiation from a 450 W medium-pressure mercury lamp in an ACE Glass photochemistry cabinet. All co-crystals were finely ground using a mortar and pestle, and then placed between a pair of Pyrex glass plates. Samples were irradiated in six-hour intervals.

Synthesis of trans-1-(2,3,4,5,6-pentafluorophenyl)-2-(2,3,5,6-tetrafluorophenyl)ethylene (**9F**):

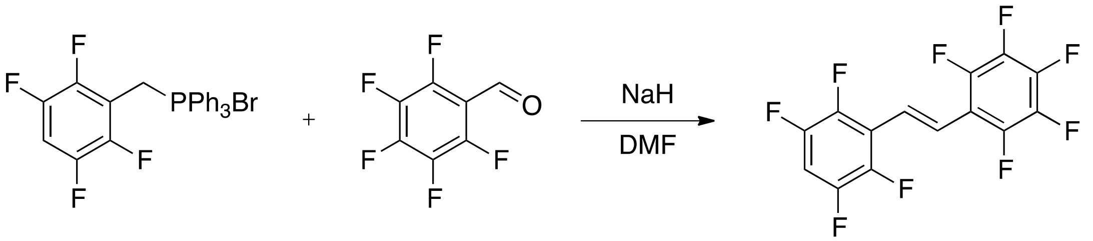

Triphenyl-(2,3,,5,6-tetrafluorobenzyl)phosphonium bromide (2.67 g, 5.3 mmol) and NaH (165 mg, 6.9 mmol) were stirred in 13 mL of DMF for 15 min. 2,3,4,5,6- pentafluorobenzaldehyde (1.1 g, 5.6 mmol) was then added to the mixture and the solution is stirred at room temperature. After 12 h, the reaction is poured on H_2O and the white solid was filtered. Column chromatography using a 1:1 CH_2Cl_2: *n*-hexane system afforded *trans*-1-(2,3,4,5,6-pentafluorophenyl)-2-(2,3,5,6-tetrafluorophenyl)ethylene (**9F**) as a white crystalline solid (1.35 g, 3.95 mmol, 75% yield): ^{1}H NMR (300 MHz, DMSO-d_6) δ 7.95 (tt, $^2J_{HF}$ = 10.5, $^3J_{HF}$ = 7.8 Hz, 1H), 7.35-7.29 (d, $^2J_{HH}$ = 18 Hz, 1H), 7.29-7.23 (d, $^2J_{HH}$ = 18 Hz, 1H).

Synthesis of the co-crystal (**SB**)·(**8F**) *and the cross-photoproduct* (**SB-8F-cb**).

Single crystals of (**SB**)·(**8F**) were obtained by combining a solution of **8F** (25 mg) in toluene (1 mL) with a solution of **SB** (14 mg, 1:1 molar ratio) in of toluene (1 mL). Single crystals suitable for X-ray diffraction studies were realized within 2 days after the slow evaporation of toluene. The photoreaction reached a quantitative yield within 30 hours of irritation, as determined by ^{1}H NMR spectroscopy. Single crystals of **SB-8F-cb**, suitable for X-ray diffraction, were obtained within

one day after the slow evaporation of a clear solution of the photoproduct (35 mg) in ethanol (5 mL).

Synthesis of the co-crystal (**BPE**)·(**8F**) *and the cross-photoproduct* (**BPE-8F-cb**).

Single crystals of (**BPE**)·(**8F**) were obtained by combining a solution of **8F** (25 mg) in toluene (1 mL) with a solution of **BPE** (14 mg, 1:1 molar ratio) in of toluene (1 mL). Single crystals suitable for X-ray diffraction studies were realized within 1 day after the slow evaporation of toluene. The photoreaction reached a quantitative yield within 30 hours of irritation, as determined by ^{1}H NMR spectroscopy. Single crystals of **BPE-8F-cb**, suitable for X-ray diffraction, were obtained within one day after the slow evaporation of a clear solution of the photoproduct (35 mg) in ethanol (3 mL).

Synthesis of the co-crystal (**SBZ**)·(**8F**) *and the cross-photoproduct* (**SBZ-8F-cb**).

Single crystals of (**SBZ**)·(**8F**) were obtained by combining a solution of **8F** (25 mg) in toluene (1 mL) with a solution of **SBZ** (14 mg, 1:1 molar ratio) in of ethanol (1 mL). Single crystals suitable for X-ray diffraction studies were realized within 1 day after the slow evaporation of toluene. The photoreaction reached a quantitative yield within 30 hours of irritation, as determined by ^{1}H NMR spectroscopy. Single crystals of **SBZ-8F-cb**, suitable for X-ray diffraction, were obtained within one day after the slow evaporation of a clear solution of the photoproduct (34 mg) in a toluene:ethanol mixture (2 mL, 1:1, v:v).

Synthesis of the co-crystal (**SBZ**)·(**9F**) *and the cross-photoproduct* (**SBZ-9F-cb**).

Single crystals of (**SBZ**)·(**9F**) were obtained by combining a solution of **9F** (25 mg) in toluene (2 mL) with a solution of **SBZ** (13 mg, 1:1 molar ratio) in of toluene (1 mL). Single crystals suitable for X-ray diffraction studies were realized within 1 day after the slow evaporation of toluene. The photoreaction reached a quantitative yield within 30 hours of irritation, yielding **SBZ-9F-cb** as a light brown solid, which was determined by ^{1}H NMR spectroscopy. Single crystals of [H-(**SBZ-9F-cb**)][***p*-TsO**], suitable for X-ray diffraction, were obtained within one day after the slow evaporation of a clear solution of **SBZ-9F-cb** (20 mg) and *p*-TsOH (7.3 mg) in a dichloromethane:methanol mixture (2 mL, 1:1, v:v).

S2. ¹H NMR Spectral Data.

^{1}H-NMR spectra were recorded using a Bruker AVANCE-400 NMR spectrometer operating at 400 MHz using DMSO-d_6 as the NMR solvent. All data were processed with MestReNova suite of software programs.

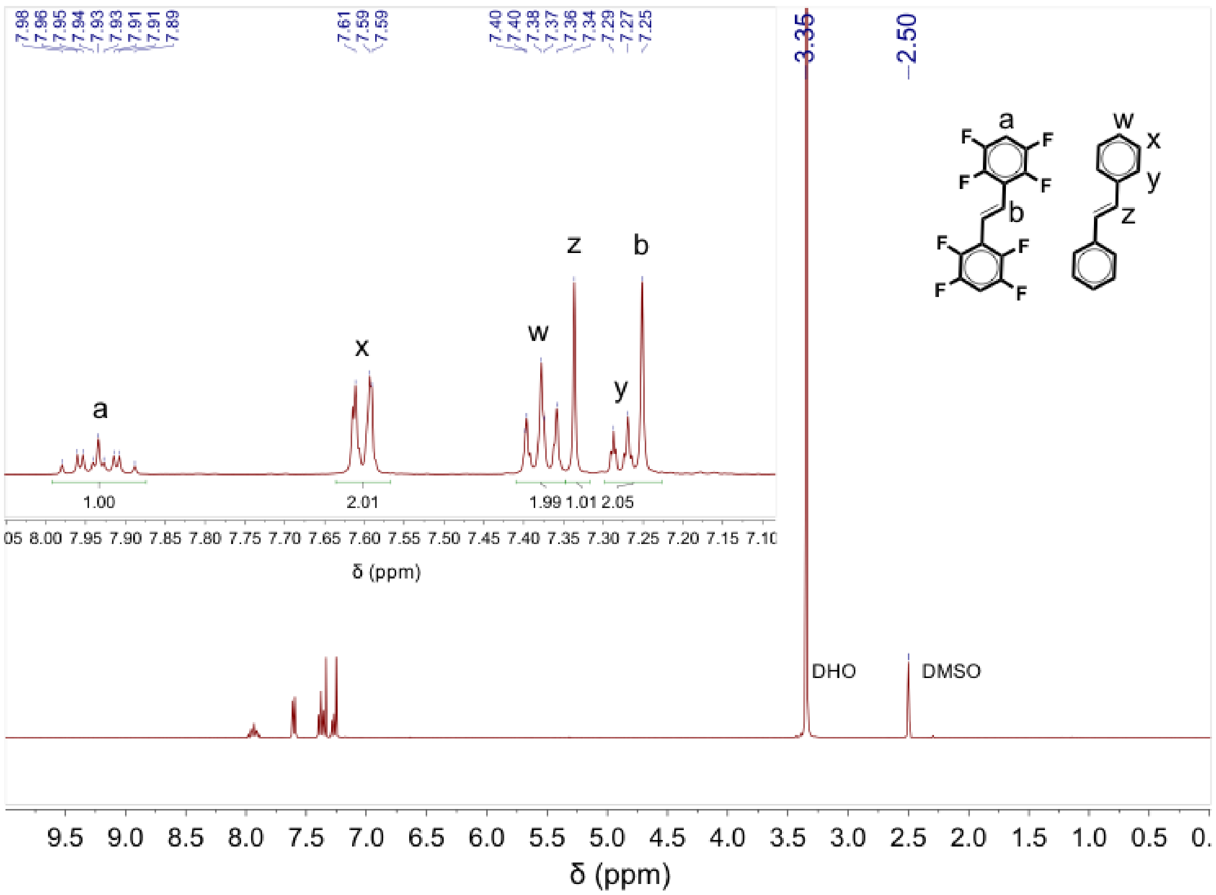

Figure S1. ^{1}H NMR spectra of (**SB**)·(**8F**).

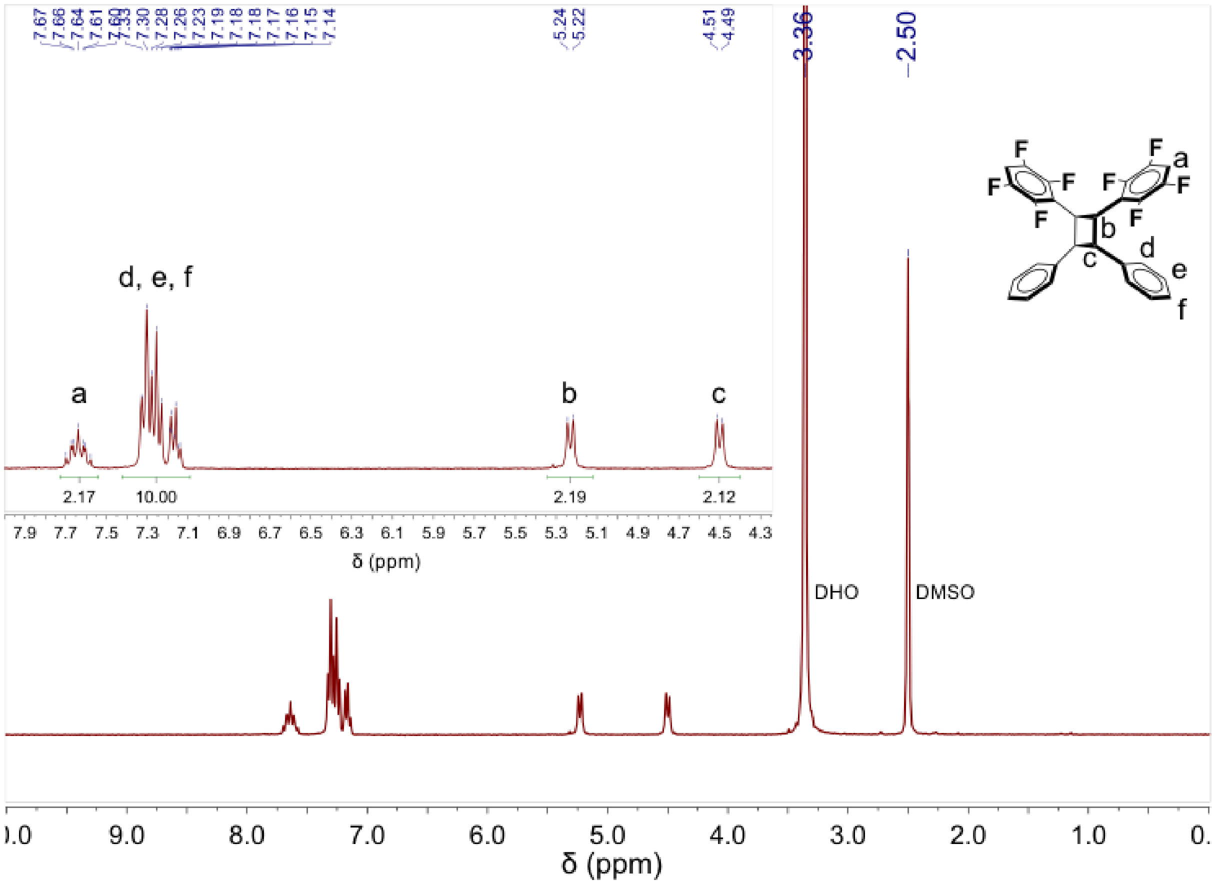

Figure S2. ^{1}H NMR spectra of **SB-8F-cb**.

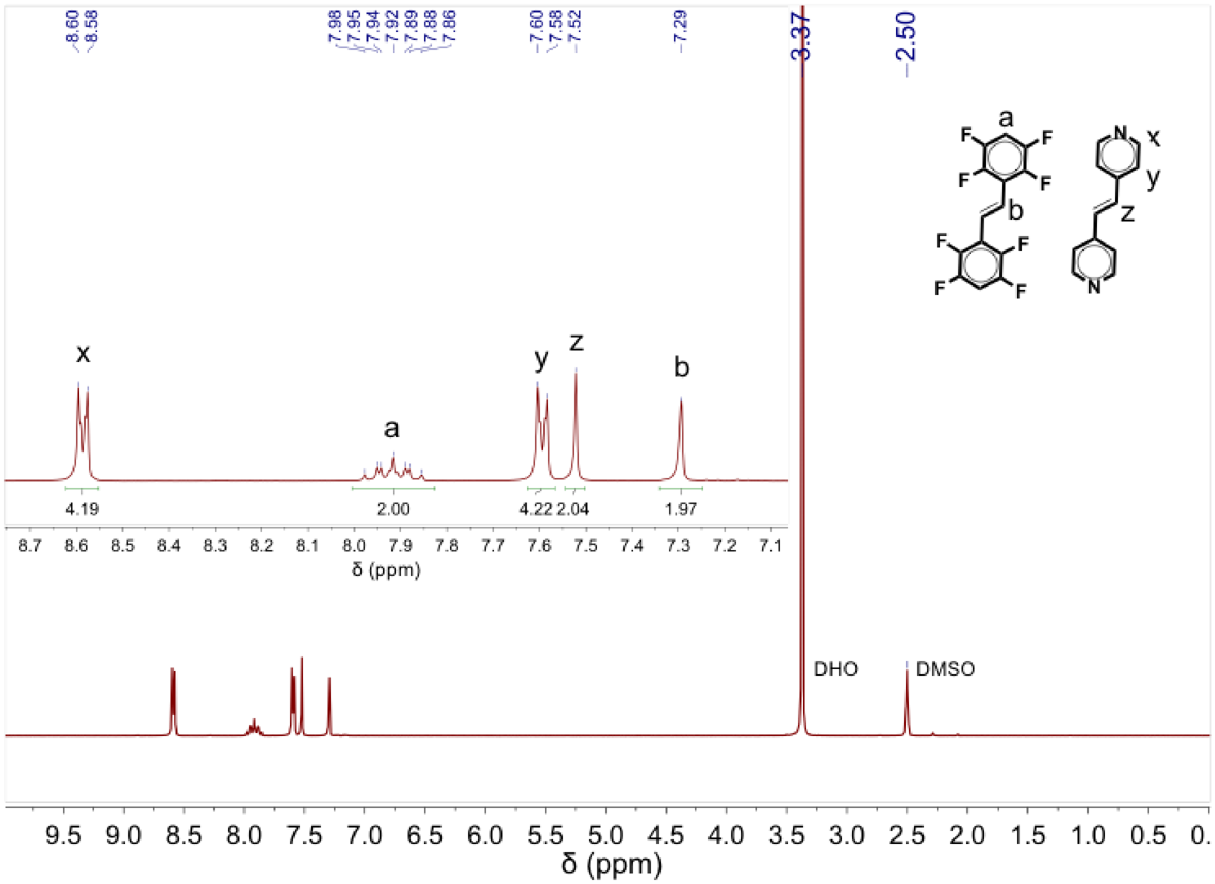

Figure S3. ^{1}H NMR spectra of (**BPE**)·(**8F**).

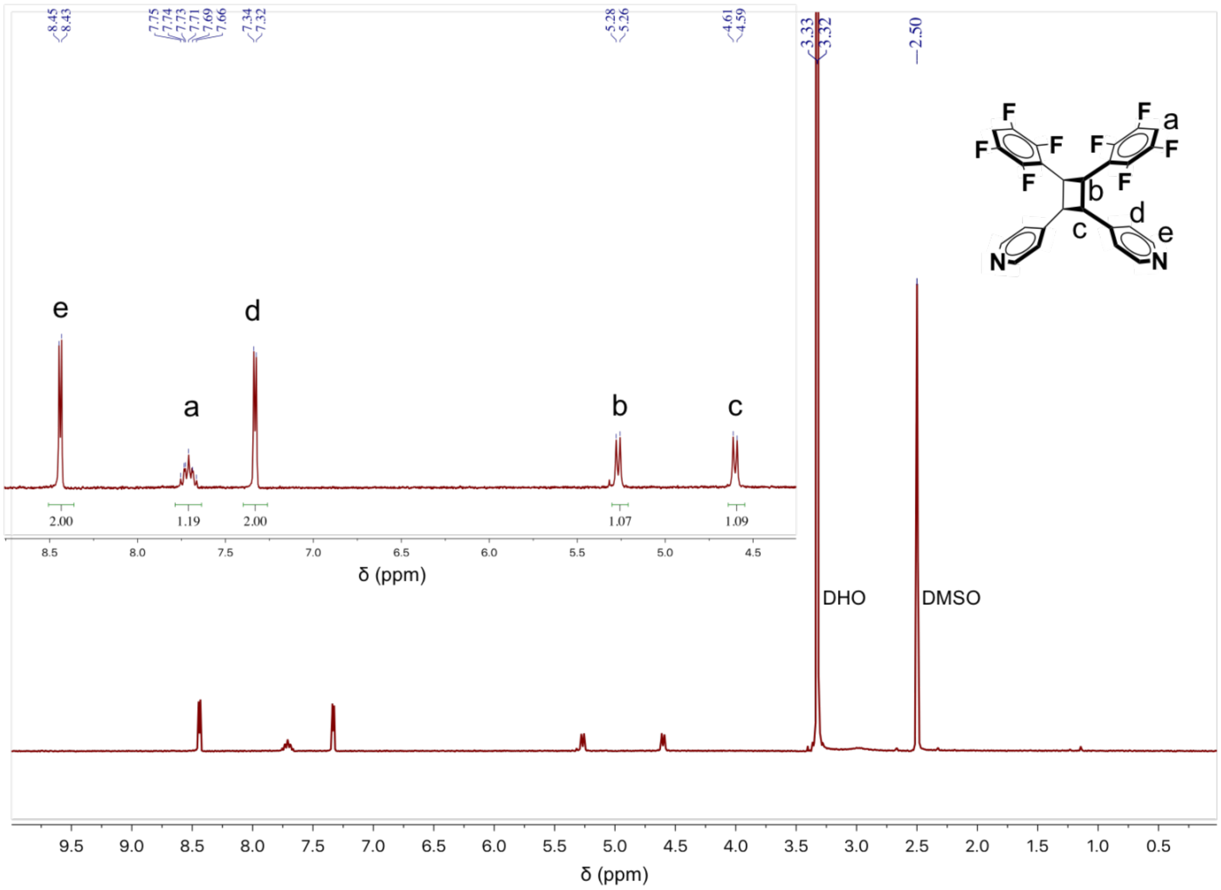

Figure S4. ^{1}H NMR spectra of (**BPE-8F-cb**).

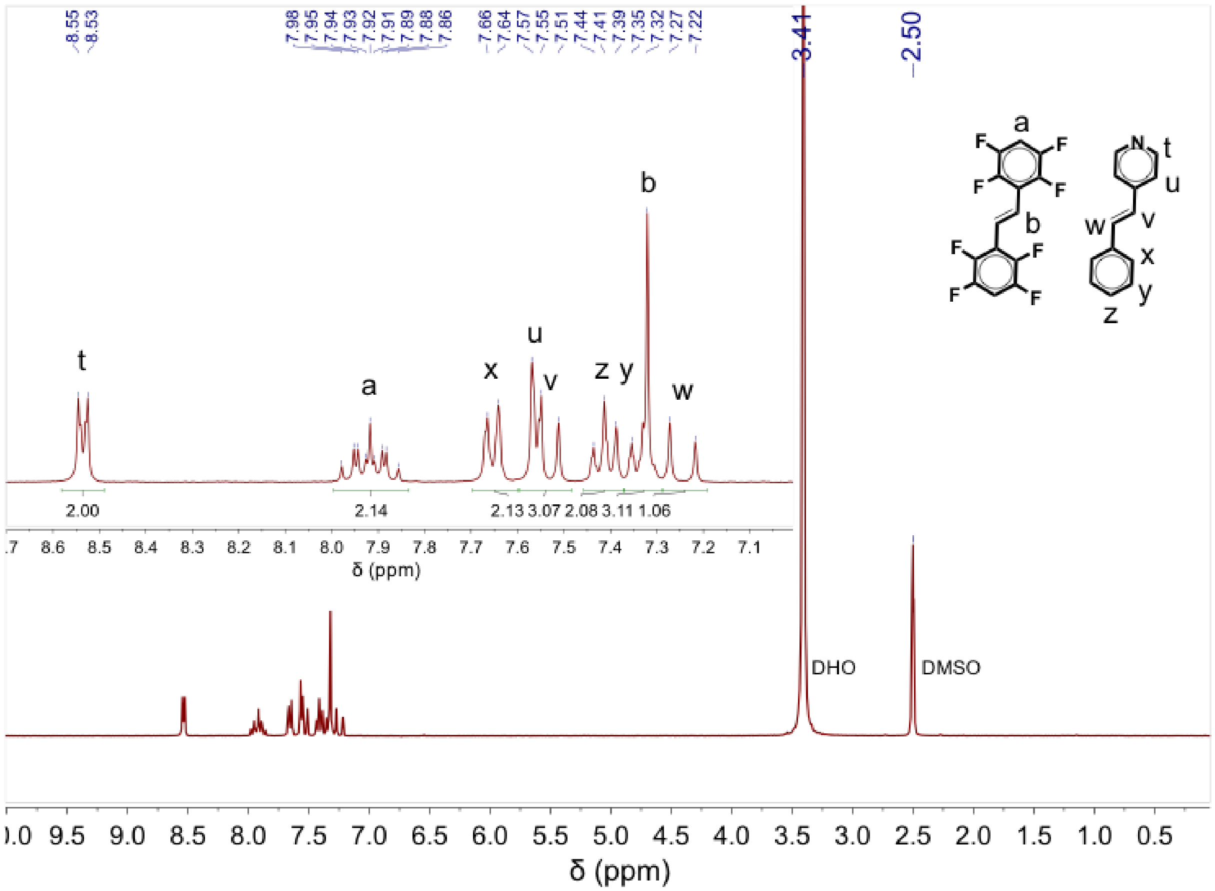

Figure S5. ^{1}H NMR spectra of (**SBZ**)·(**8F**).

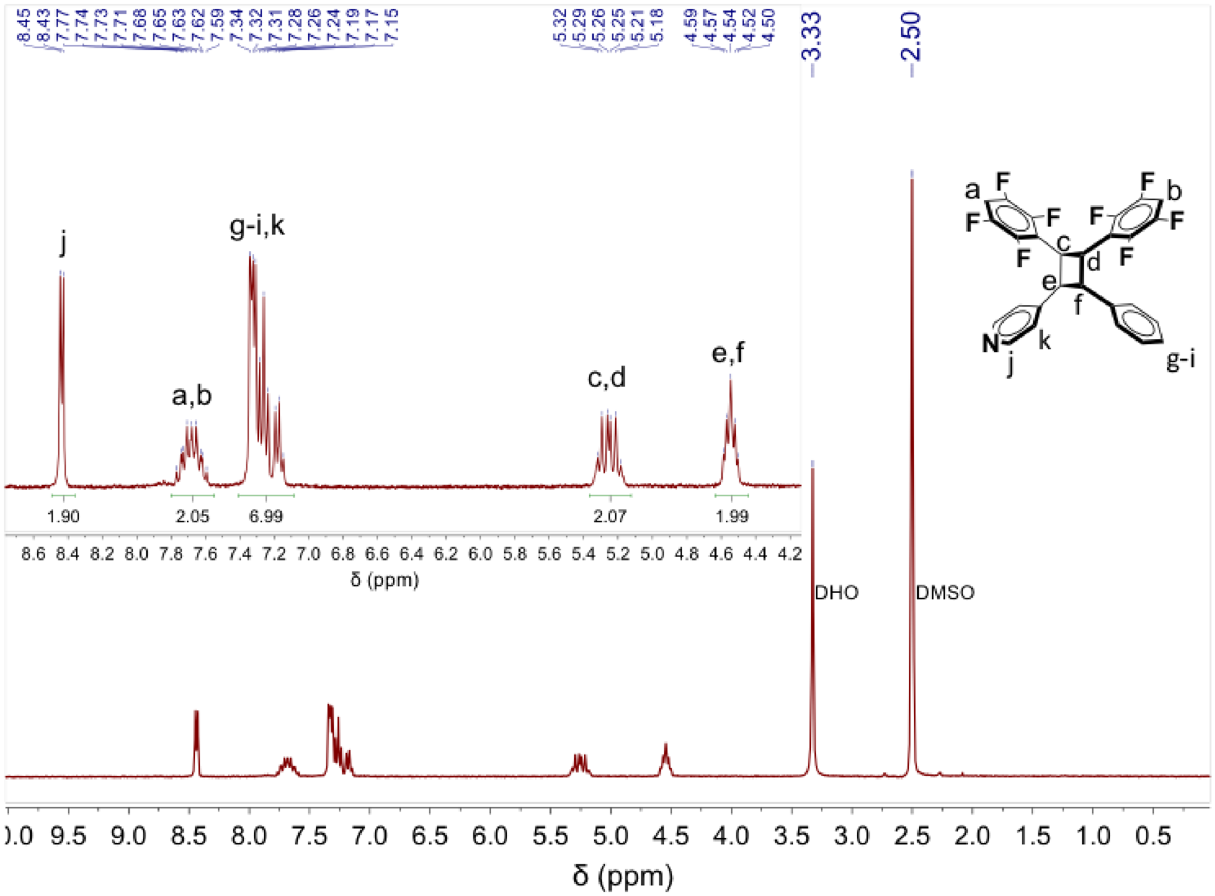

Figure S6. ^{1}H NMR spectra of **SBZ-8F-cb**.

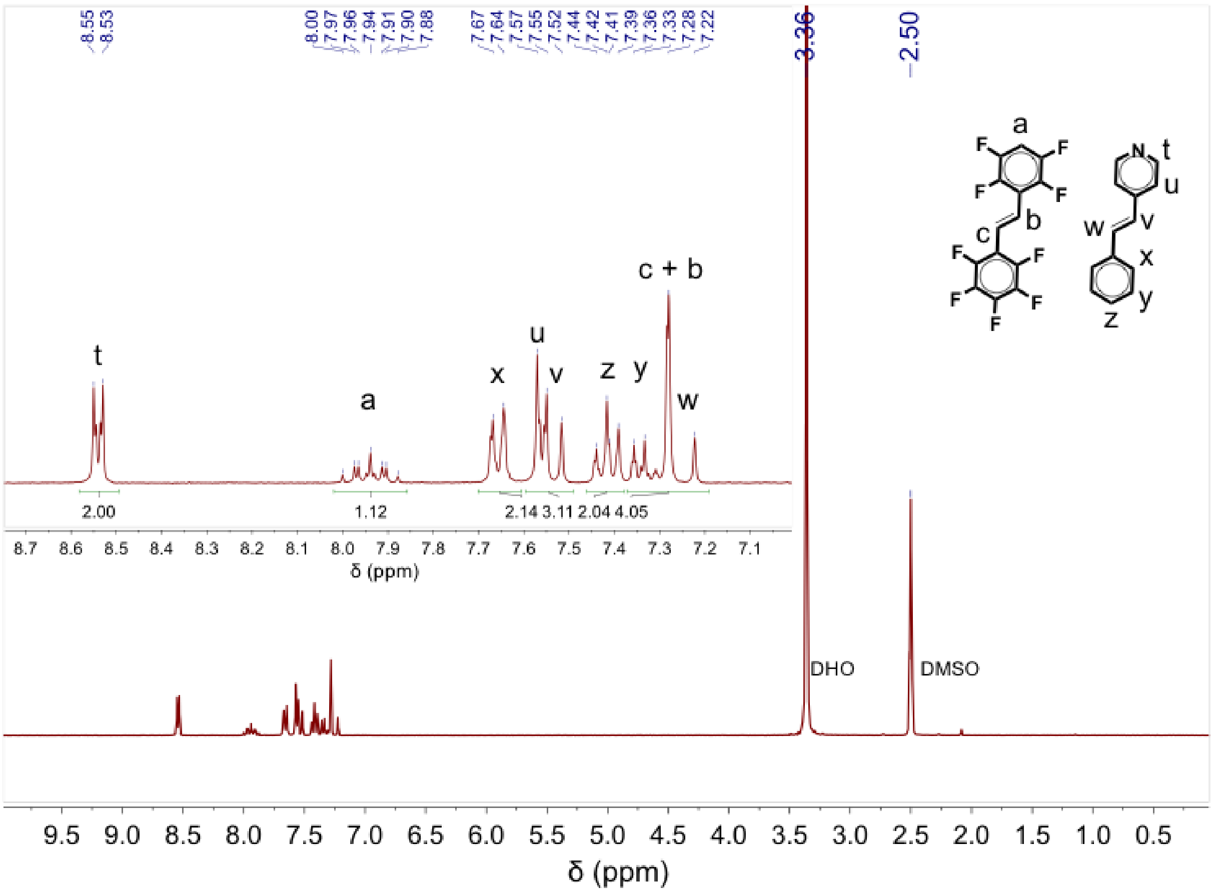

Figure S7. ^{1}H NMR spectra of (**SBZ**)·(**9F**).

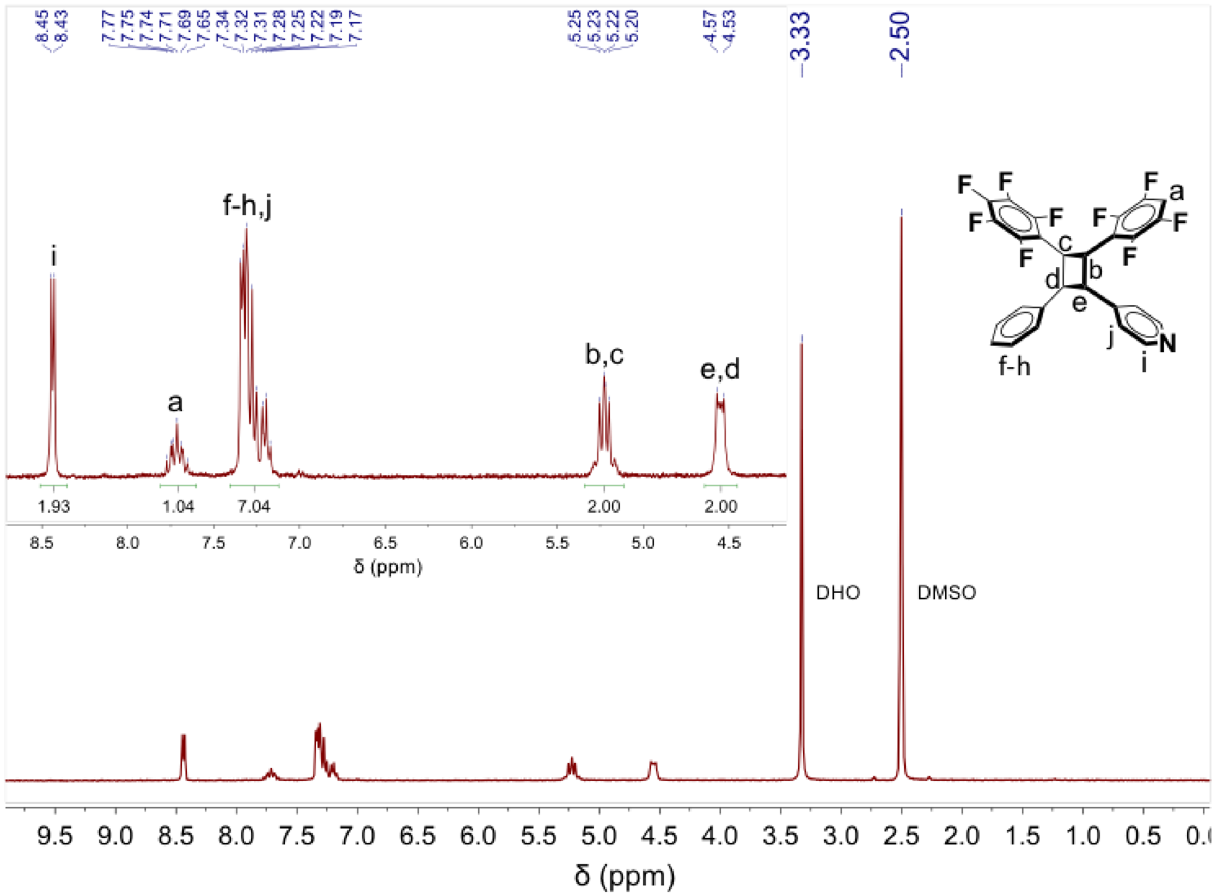

Figure S8. ^{1}H NMR spectra of **SBZ-9F-cb**.

S3. Powder X-ray Diffraction Data

PXRD data were collected from samples mounted on glass slides by a Bruker D8 DaVinci powder X-ray diffraction (XRD) system using CuK$_{\alpha}$1 radiation (λ = 1.54056 Å) (scan type: locked coupled; scan mode: continuous; step size: 0.02°).

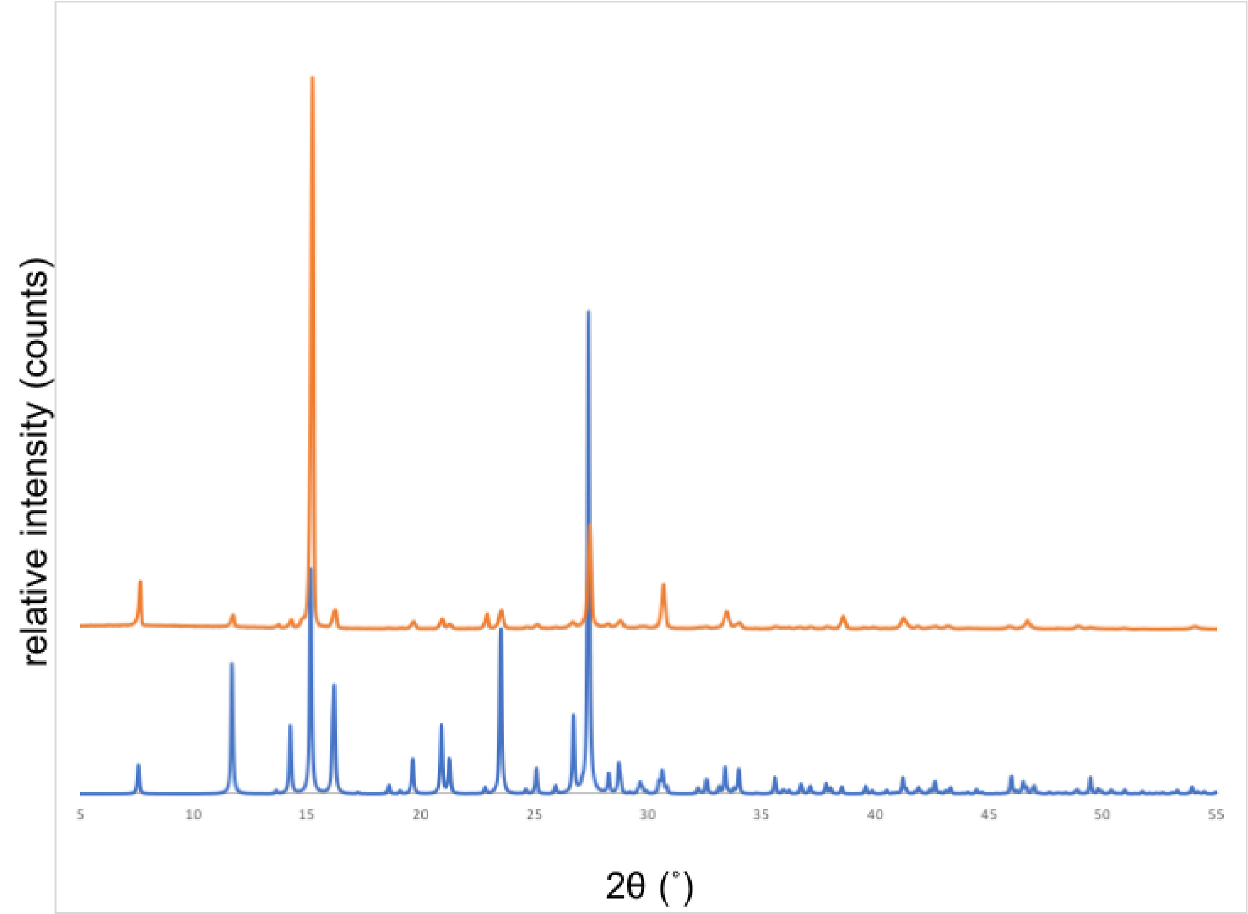

Figure S9. Powder X-ray diffraction of powder of (**SB**)·(**8F**) and simulated pattern from single-crystal X-ray diffraction data.

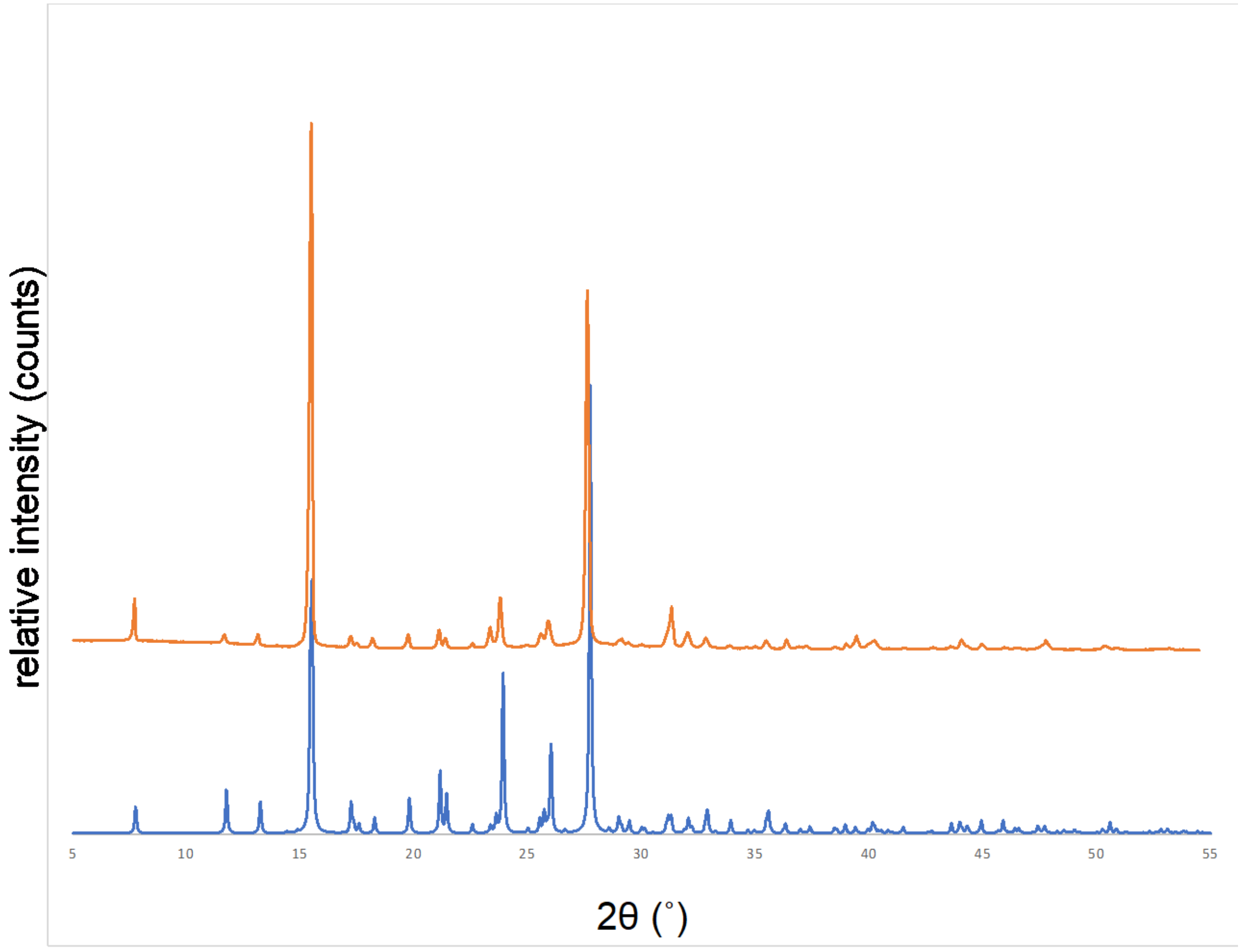

Figure S10. Powder X-ray diffraction of powder of (**BPE**)·(**8F**) and simulated pattern from single-crystal X-ray diffraction data.

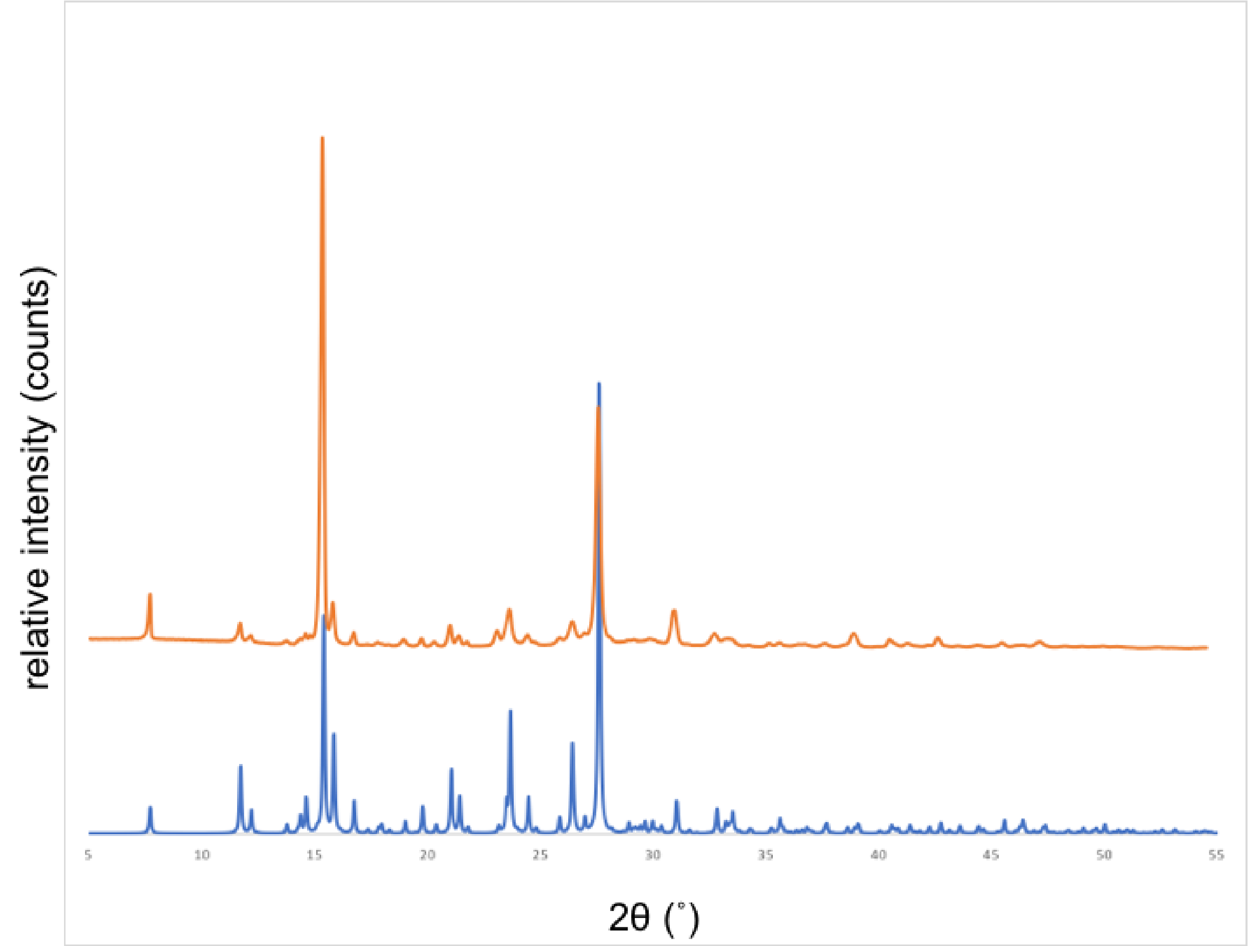

Figure S11. Powder X-ray diffraction of powder of (**SBZ**)·(**8F**) and simulated pattern from single-crystal X-ray diffraction data.

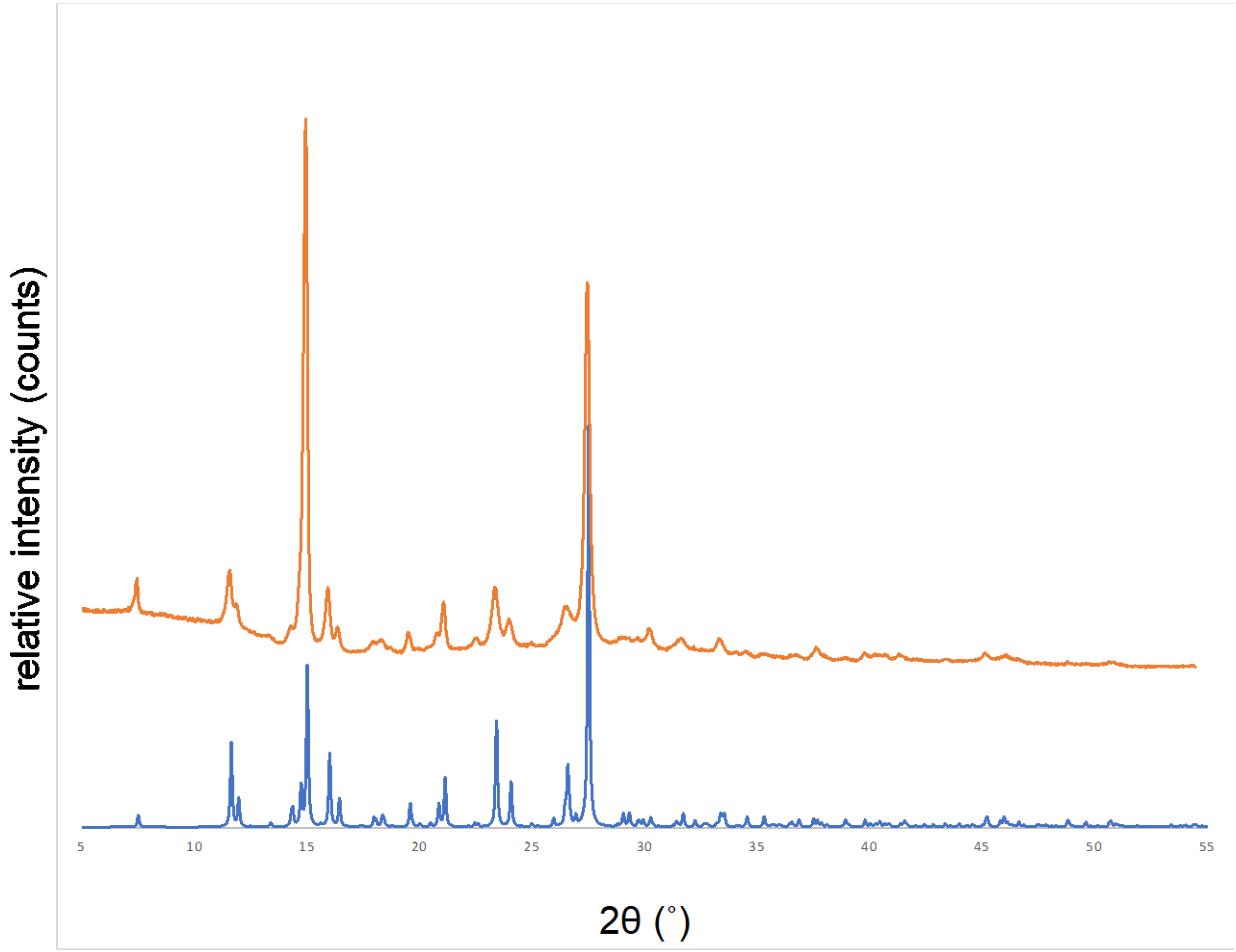

Figure S12. Powder X-ray diffraction of powder of (**SBZ**)·(**9F**) and simulated pattern from single-crystal X-ray diffraction data.

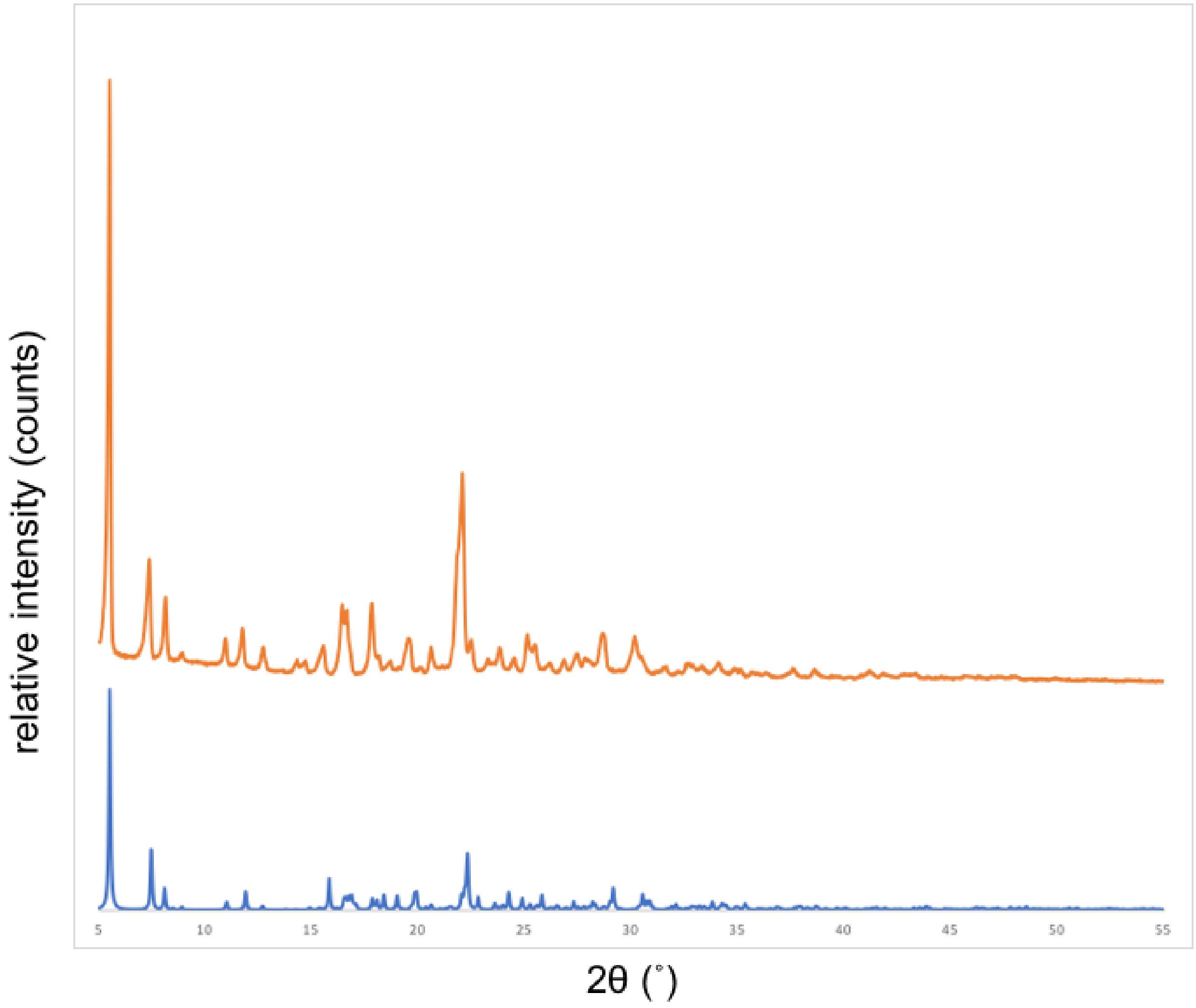

Figure S13. Powder X-ray diffraction of powder of [H-**SBZ-9F-cb**][***p*-TsO**] and simulated pattern from single-crystal X-ray diffraction data.

S4. Single Crystal X-ray Diffraction Data

Single crystal data was collected with a Bruker APEX II Kappa Diffractometer equipped with an Oxford Cryostream low temperature device using MoK_{α} radiation (λ = 0.71073 Å) after having been secured to Mitegen magnetic mounts using Paratone oil. Data collection strategies to ensure maximum data redundancy and completeness were calculated using Apex2.[4] All calculation dealing with data collection, initial indexing, frame integration, Lorentz- polarization corrections and final cell parameter were again carried out by Apex2.[5] All structures were solved using ShelXT[6] and refined using ShelXL[7] in the Olex2[8] graphical user interface. Hydrogen atoms associated with carbon atoms were refined in geometrically constrained positions with isotropic thermal parameter $U_{iso}(H) = 1.2\ U_{eq}(C_{CH})$.

Table S1. Crystal data and structure refinement for (**SB**)·(**8F**) and **SB-8F-cb**.

	**(SB)·(8F)**	**SB-8F-cb**
CCDC	2042036	2042037
Empirical formula	$C_{28}H_{16}F_8$	$C_{28}H_{16}F_8$
Formula weight	504.41	504.41
Temperature/K	296.15	100.04
Crystal system	triclinic	triclinic
Space group	P-1	P-1
a/Å	6.2588(4)	8.0657(3)
b/Å	7.6417(6)	10.9450(4)
c/Å	11.6953(8)	12.9478(5)
α/°	87.276(4)	68.754(2)
β/°	89.359(4)	87.400(2)
γ/°	82.433(4)	78.137(2)
Volume/$Å^3$	553.86(7)	1042.05(7)
Z	1	2
$\rho_{calc}g/cm^3$	1.512	1.608
μ/mm^{-1}	0.134	0.143
F(000)	256.0	512.0
Crystal size/mm^3	0.547 × 0.529 × 0.053	0.584 × 0.316 × 0.253
Radiation	MoKα (λ = 0.71073)	MoKα (λ = 0.71073)
2Θ range for data collection/°	3.486 to 50.894	3.376 to 61.366
Index ranges	-7 ≤ h ≤ 7, -9 ≤ k ≤ 9, -14 ≤ l ≤ 14	-11 ≤ h ≤ 11, -15 ≤ k ≤ 15, -18 ≤ l ≤ 14
Reflections collected	8790	16630
Independent reflections	2034 [R_{int} = 0.0252, R_{sigma} = 0.0260]	6175 [R_{int} = 0.0245, R_{sigma} = 0.0362]
Data/restraints/parameters	2034/0/163	6175/0/325
Goodness-of-fit on F^2	1.024	1.057
Final R indexes [I>=2σ (I)]	R_1 = 0.0522, wR_2 = 0.1378	R_1 = 0.0617, wR_2 = 0.1568
Final R indexes [all data]	R_1 = 0.0858, wR_2 = 0.1733	R_1 = 0.0851, wR_2 = 0.1741
Largest diff. peak/hole / e $Å^{-3}$	0.29/-0.23	0.63/-0.26

Table S2. Crystal data and structure refinement for (**BPE**)·(**8F**) and **BPE-8F-cb**.

	**(BPE)·(8F)**	**BPE-8F-cb**
CCDC	2042038	2042039
Empirical formula	$C_{26}H_{14}F_8N_2$	$C_{26}H_{14}F_8N_2$
Formula weight	506.39	506.39
Temperature/K	273.15	293(2)
Crystal system	triclinic	triclinic
Space group	P-1	P-1
a/Å	6.286(3)	8.1788(6)
b/Å	7.690(4)	10.5773(8)
c/Å	11.654(7)	12.7786(10)
α/°	81.58(4)	108.775(4)
β/°	81.41(4)	99.388(4)
γ/°	81.23(4)	99.387(4)
Volume/Å^3	546.1(5)	1004.75(13)
Z	1	2
ρ_{calc}g/cm^3	1.540	1.674
μ/mm^{-1}	0.139	0.151
F(000)	256.0	512.0
Crystal size/mm^3	0.369 × 0.217 × 0.054	0.271 × 0.164 × 0.142
Radiation	MoKα (λ = 0.71073)	MoKα (λ = 0.71073)
2Θ range for data collection/°	3.564 to 52.43	3.46 to 55.07
Index ranges	$-7 \le h \le 7$, $-9 \le k \le 9$, $-14 \le l \le 14$	$-10 \le h \le 10$, $-13 \le k \le 13$, $-16 \le l \le 16$
Reflections collected	11212	15472
Independent reflections	2147 [R_{int} = 0.0527, R_{sigma} = 0.0424]	4521 [R_{int} = 0.0375, R_{sigma} = 0.0501]
Data/restraints/parameters	2147/0/163	4521/0/325
Goodness-of-fit on F^2	0.935	1.057
Final R indexes [I>=2σ (I)]	R_1 = 0.0529, wR_2 = 0.1492	R_1 = 0.0498, wR_2 = 0.1239
Final R indexes [all data]	R_1 = 0.1116, wR_2 = 0.1965	R_1 = 0.0852, wR_2 = 0.1423
Largest diff. peak/hole / e Å^{-3}	0.26/-0.20	0.38/-0.27

Table S3. Crystal data and structure refinement for (**SBZ**)·(**8F**) and **SBZ-8F-cb**.

	**(SBZ)·(8F)**	**SBZ-8F-cb**
CCDC	2042040	2042041
Empirical formula	$C_{27}H_{15}F_8N$	$C_{27}H_{15}F_8N$
Formula weight	505.40	505.40
Temperature/K	299.79	99.97
Crystal system	triclinic	triclinic
Space group	P-1	P-1
a/Å	6.2524(8)	8.0271(8)
b/Å	7.6581(12)	10.9328(12)
c/Å	23.133(3)	12.7798(13)
α/°	87.316(9)	68.901(6)
β/°	85.878(9)	87.057(6)
γ/°	81.898(9)	78.538(6)
Volume/Å^3	1093.0(3)	1025.23(19)
Z	2	2
ρ_{calc}g/cm^3	1.536	1.637
μ/mm^{-1}	0.138	0.147
F(000)	512.0	512.0
Crystal size/mm^3	0.432 × 0.343 × 0.153	0.234 × 0.171 × 0.079
Radiation	MoKα (λ = 0.71073)	MoKα (λ = 0.71073)
2Θ range for data collection/°	3.532 to 54.204	3.416 to 54.35
Index ranges	-8 ≤ h ≤ 8, -9 ≤ k ≤ 9, -29 ≤ l ≤ 29	-10 ≤ h ≤ 10, -14 ≤ k ≤ 11, -16 ≤ l ≤ 16
Reflections collected	20678	14002
Independent reflections	4748 [R_{int} = 0.0293, R_{sigma} = 0.0266]	4499 [R_{int} = 0.0405, R_{sigma} = 0.0650]
Data/restraints/parameters	4748/0/325	4499/0/325
Goodness-of-fit on F^2	1.024	0.995
Final R indexes [I>=2σ (I)]	R_1 = 0.0517, wR_2 = 0.1495	R_1 = 0.0600, wR_2 = 0.1385
Final R indexes [all data]	R_1 = 0.0968, wR_2 = 0.1862	R_1 = 0.1098, wR_2 = 0.1649
Largest diff. peak/hole / e Å^{-3}	0.28/-0.18	0.60/-0.29

Table S4. Crystal data and structure refinement for (**SBZ**)·(**9F**) and [H-(**SBZ-9F-cb)**][***p*-TsO**].

	(**SBZ**)·(**9F**)	[H-(**SBZ-9F-cb)**][***p*-TsO**]
CCDC	2042042	2042043
Empirical formula	$C_{27}H_{14}F_9N$	$C_{34}H_{22}F_9NO_3S$
Formula weight	523.39	695.58
Temperature/K	296.15	99.99
Crystal system	triclinic	monoclinic
Space group	P-1	$P2_1/n$
a/Å	6.2122(8)	5.8302(3)
b/Å	7.6774(8)	23.7785(12)
c/Å	23.672(3)	22.0776(11)
α/°	85.610(7)	90
β/°	87.631(5)	96.947(2)
γ/°	82.946(7)	90
Volume/Å^3	1116.6(2)	3038.2(3)
Z	2	4
ρ_{calc}g/cm^3	1.557	1.521
μ/mm^{-1}	0.144	0.2
F(000)	528	1416
Crystal size/mm^3	0.34 × 0.3 × 0.025	0.1 × 0.03 × 0.01
Radiation	Mo Kα (λ = 0.71073)	MoKα (λ = 0.71073)
2Θ range for data collection/°	5.18 to 50.798	5.466 to 53.732
Index ranges	-7 ≤ h ≤ 7, -9 ≤ k ≤ 8, -27 ≤ l ≤ 28	-7 ≤ h ≤ 6, -30 ≤ k ≤ 30, -28 ≤ l ≤ 28
Reflections collected	5729	97040
Independent reflections	3871 [R_{int} = 0.0525, R_{sigma} = 0.1477]	6532 [R_{int} = 0.0858, R_{sigma} = 0.0289]
Data/restraints/parameters	3871/0/334	6532/0/434
Goodness-of-fit on F^2	0.972	1.037
Final R indexes [I>=2σ (I)]	R_1 = 0.0639, wR_2 = 0.1110	R_1 = 0.0399, wR_2 = 0.0866
Final R indexes [all data]	R_1 = 0.2505, wR_2 = 0.1696	R_1 = 0.0608, wR_2 = 0.0944
Largest diff. peak/hole / e Å^{-3}	0.18/-0.19	0.29/-0.37

S5. Molecular Modeling

Electrostatic potentials for each functionalized alkene were calculated at ground state in gas phase using Spartan ’18 V1.2.0 software. Equilibrium geometry was used for calculation without further constraints. All calculations were performed using the B3LYP/6-31G* density functional model.

Table S5. Mulliken charge distribution values along with the designated number system.

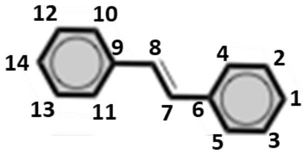

Template	Energy (a.u.)	Mulliken Charges
**SB**	-540.71	C1(C14): -0.129; H1(H14): 0.130<br>C2(C13): -0.131; H2(H13): 0.130<br>C3(C12): -0.129; H3(H12): 0.130<br>C4(C11): -0.180; H4(H11): 0.130<br>C5(C10): -0.198; H5(H10): 0.127<br>C6(C9): 0.182<br>C7(C8): -0.189; H7(H8): 0.127
**BPE**	-572.78	N1(N14): -0.407<br>C2(C13): 0.036; H2(H13): 0.144<br>C3(C12): 0.040; H3(H12): 0.137<br>C4(C11): -0.185; H4(H11): 0.137<br>C5(C10): -0.203; H5(H10): 0.137<br>C6(C9): 0.193<br>C7(C8): -0.174; H7 (H8): 0.138
**SBZ**	-556.75	N1: -0.411<br>C2: 0.039; H2: 0.140<br>C3: 0.034; H3: 0.140<br>C4: -0.205; H4: 0.132<br>C5: -0.187; H5: 0.134<br>C6: 0.196<br>C7: -0.183; H7: 0.132<br>C8: -0.180; H8: 0.133<br>C9: 0.179<br>C10: -0.177; H10: 0.133<br>C11: -0.195; H11:0.131<br>C12: -0.131; H12: 0.134<br>C13: -0.130; H13: 0.135<br>C14: -0.127; H14: 0.134

**8F**	-1334.54	C1(C14): -0.293; H1(H14): 0.182<br>C2(C13): 0.346; F2(F13): -0.279<br>C3(C12): 0.344; F3(F12): -0.279<br>C4(C11): 0.262; F4(F11): -0.273<br>C5(C10): 0.235; F5(F10): 0.272<br>C6(C9): 0.039<br>C7(C8): -0.190; H7(H8): 0.178
**9F**	-1433.76	C1: -0.293; H1: 0.243<br>C2: 0.344; F2: -0.278<br>C3: 0.346; F3: -0.279<br>C4: 0.235; F4: -0.272<br>C5: 0.261; F5: -0.273<br>C6: 0.039<br>C7: -0.191; H7: 0.176<br>C8: -0.189; H8: 0.178<br>C9: 0.040<br>C10: 0.240; F10: -0.269<br>C11: 0.270; F11: -0.270<br>C12: 0.263; F12: -0.264<br>C13: 0.265; F13: -0.265<br>C14: 0.262; F14: -0.259

S6. Supplementary References

1. Baldrighi, M.; Metrangolo, P.; Meyer, F.; Pilati, T.; Proserpio, D.; Resnati, G.; Terraneo, G. *J. Fluorine Chem.* **2010,** *131* (11), 1218-1224.
2. Sinnwell, M. A.; Ingenthron, B. J.; Groeneman, R. H.; MacGillivray, L. R. *J. Fluorine Chem.* **2016,** *188*, 5-9.
3. Efange, S. M. N.; Michelson, R. H.; Remmel, R. P.; Boudreau, R. J.; Dutta, A. K.; Freshler, A. *J. Med. Chem.* **1990,** *33* (12), 3133-3138.
4. Bruker (2012). APEX2. Bruker AXS Inc., Madison, Wisconsin, USA.
5. Bruker (2012). APEX2. Bruker AXS Inc., Madison, Wisconsin, USA.
6. Sheldrick, G. *Acta Crystallographica Section A* **2015,** *71* (1), 3-8.
7. Sheldrick, G. *Acta Crystallographica Section C* **2015,** *71* (1), 3-8.
8. Dolomanov, O. V.; Bourhis, L. J.; Gildea, R. J.; Howard, J. A. K.; Puschmann, H. *J. Appl. Crystallogr.* **2009,** *42* (2), 339-341.
