# Supplementary material for: Supramolecular construction of a cyclobutane ring system with four different substituents in the solid state
Source: Commun Chem. 2021 May 10;4:60. doi: 10.1038/s42004-021-00493-3 (PMC9814370; doi:10.1038/s42004-021-00493-3)
Supplement: Supplementary file 10 — Description of Additional Supplementary Files [file 42004_2021_493_MOESM10_ESM.pdf]

## Description of Additional Supplementary Files

**File Name:** Supplementary Data 1

**Description**: SB·8F (CCDC 2042036)

**File Name:** Supplementary Data 2

**Description**: SB-8F-cb (CCDC 2042037)

**File Name:** Supplementary Data 3

**Description**: BPE·8F (CCDC 2042038)

**File Name:** Supplementary Data 4

**Description**: BPE-8F-cb (CCDC 2042039)

**File Name:** Supplementary Data 5

**Description**: SBZ·8F (CCDC 2042040)

**File Name:** Supplementary Data 6

**Description**: SBZ-8F-cb (CCDC 204041)

**File Name:** Supplementary Data 7

**Description**: SBZ·9F (CCDC 204042)

**File Name:** Supplementary Data 8

**Description**: [H-SBZ-9F-cb][p-TsO] (CCDC 204043)
